# Application of a capsaicin rinse in the treatment of burning mouth syndrome

**DOI:** 10.4317/medoral.17219

**Published:** 2011-07-15

**Authors:** Francisco-Javier Silvestre, Javier Silvestre-Rangil, Carmen Tamarit-Santafé, Daniel Bautista

**Affiliations:** 1 Assistant Professor, Department of Stomatology, University of Valencia. Head of the Stomatology Unit, Dr. Peset University Hospital. Valencia; 2 Dental surgeon, private practice; 3 Associate Professor of Odontology, Special Patients. University of Cardenal Herrera-CEU Dental School. Valencia; 4 Staff physician, Department of Preventive Medicine, Dr. Peset University Hospital. Valencia (Spain)

## Abstract

Objective: To examine the efficacy of a new topical capsaicin presentation as an oral rinse in improving the symptoms of burning mouth syndrome (BMS). Study design: A prospective, double-blind, cross-over study was made of 30 patients with BMS. There were 7 dropouts; the final study series thus comprised 23 individuals. The patients were randomized to two groups: (A) capsaicin rinse (0.02%) or (B) placebo rinse, administered during one week. After a one-week washout period, the patients were then assigned to the opposite group. Burning discomfort was scored using a visual analog scale (VAS): in the morning before starting the treatment, in the afternoon on the first day of treatment, and at the end of the week of treatment in the morning and in the afternoon. The same scoring sequence was again applied one week later with the opposite rinse. Results: The mean patient age was 72.65 ± 12.10 years, and the duration of BMS was 5.43 ± 3.23 years on average. Significant differences in VAS score were recorded in the capsaicin group between baseline in the morning (AM1) or afternoon (AA1) and the end of the week of treatment (AA7)(p=0.003 and p=0.002, respectively). Conclusion: The topical application of capsaicin may be useful in treating the discomfort of BMS, but has some limitations.

** Key words:** Burning mouth syndrome, stomatodynia, capsaicin, treatment, clinical management.

## Introduction

Burning mouth syndrome (BMS) manifests as a burning sensation of the tongue, lips or entire oral cavity, in the absence of any objective lesions or laboratory test findings capable of accounting for the discomfort (1). Patients with BMS usually have other associated conditions such as dry mouth or taste alterations – these manifestations being common in situations of anxiety or depression (2). Most affected subjects are females, particularly postmenopausal women. The revised chronic pain classification of the International Headache Society (Headache Classification 2004) defines BMS as a burning sensation for which no medical or dental cause can be established (3).

The diagnosis of the syndrome is based on the clinical manifestations. The underlying cause is unknown, though many potentially influencing conditions have been cited, such as local, general and psychopathological factors. More recently, BMS has been associated to a neuropathic mechanism affecting peripheral or central levels of the nervous system (4-6).

Very few cases of spontaneous remission of the symptoms have been reported. The treatments proposed for controlling the discomfort caused by BMS include alpha-lipoic acid, cognitive-behavioral therapy, the administration of anticonvulsivants (particularly clonazepam), and topical capsaicin (3,7,8). However, most of the studies lack controls, the measurements are not reproducible, and the sample sizes are limited (3). 

Topical capsaicin has been used as a treatment alternative for controlling neuropathic pain. This substances induces desensitization to thermal, chemical and mechanical stimuli. It has also been used for the treatment of the discomfort caused by BMS, with positive results. Petruzzi et al. (9) showed 0.025% capsaicin via the systemic route to be effective in the context of short treatments. The drug has also been used topically, with good results (10).

The present study was designed to examine the efficacy of a new topical capsaicin presentation as an oral rinse in improving the symptoms of BMS. In addition, the possible adverse effects of such treatment were evaluated.

## Material and Methods

 -Study design

A prospective, double-blind, cross-over study was made involving randomized patient distribution to treatment. BMS was diagnosed according to the current criteria, and the discomfort had been present on a daily basis for at least 6 months (2). A visual analog scale (VAS) was used to score discomfort at different times during the study (0 cm = no discomfort, 10 cm = unbearable or maximum discomfort).

The patients were randomized to two groups: (A) 0.02% capsaicin rinse or (B) placebo, administered three times a day. The rinses were applied for about 30 seconds in volumes of 15 ml. A baseline assessment of discomfort was made in the morning before treatment in both the capsaicin group (AM1) and in the placebo group (BM1). The VAS score was then again recorded in the afternoon of the first day of treatment in both groups (AA1 and BA1) and at the end of one week of treatment in both groups, in the morning (AM7 and BM7) and in the afternoon (AA7 and BA7). After this first week of treatment, the patients completed a one-week washout period (no rinses, only regular dental hygiene in the form of tooth brushing), after which they were assigned to the opposite group for a further week of treatment. During this cross-over period, treatment and the scoring of discomfort were carried out in the same way as in the first week, but administering the opposite oral rinse. Thus, all of the patients used both rinses (capsaicin and placebo) and were scored for discomfort at the start of the study, after one week, and again after three weeks. The patients were also questioned about possible adverse effects or discomfort caused by the treatments. 

 -Patient selection

Since this was a pilot study and the number of publications on topical capsaicin for treating BMS is limited, we analyzed 23 patients (19 females and 4 males) out of a total of 30 initially planned subjects (7 dropped out in the first week of the study). The patients were randomized at the start of the first week (12 in one group and 11 in the other).

The study included patients diagnosed with BMS and with a minimum duration of the disease of 6 months. The enrolled subjects were required to use no specific medication or treatment capable of interfering with BMS, for at least one month prior to the start of the study, and written informed was obtained in all cases. On the other hand, patients with contraindications to topical capsaicin treatment were excluded from the study, as were those with serious systemic disease at the time of patient screening, patients with oral mucosal lesions that might explain the burning sensation, and subjects unable to correctly score discomfort on the VAS scale. Lastly, where indicated, those subjects who developed adverse effects were removed from the study.

The study was approved by the Clinical Research Ethics Committee of Dr. Peset University Hospital (Valencia, Spain).

 -Data analysis

The SPSS version 15.0 statistical package for Microsoft Windows was used to analyze the data. Qualitative variables were represented by their absolute (n) and relative (%) frequencies, while quantitative variables were reported as the median and range. The effects of the capsaicin rinse and placebo formulation were evaluated, comparing the distribution of the VAS scores for each rinse at the different study timepoints (AM1, AA1, BM1, BA1, AM7, AA7, BM7, BA7), based on the Wilcoxon test. Statistical significance was considered for p < 0.05.

## Results

The study initially involved 30 patients, of which 7 (23.3%) abandoned the trial in the first week of treatment. Of these 7 individuals, four belonged to the capsaicin group (2 claimed to have noted no therapeutic effect, while the other two complained of greatly increased burning sensation when using the rinse). In turn, in the control group three patients abandoned the study during the first week because of a lack of therapeutic effect.

Thus, the study finally comprised 23 patients with BMS yielding cross-over measurements of discomfort (23 in group A and 23 in group B).

The mean patient age was 72.65 ± 12.10 years (range 40-90 years). Most of the patients (65.2%) were between 70 and 80 years of age. The mean duration of the disease was 5.43 ± 3.23 years (range 1-14 years). Patients with a BMS duration of 4 and 5 years represented 39.1% of the total (n = 9).

**Figure 1:**
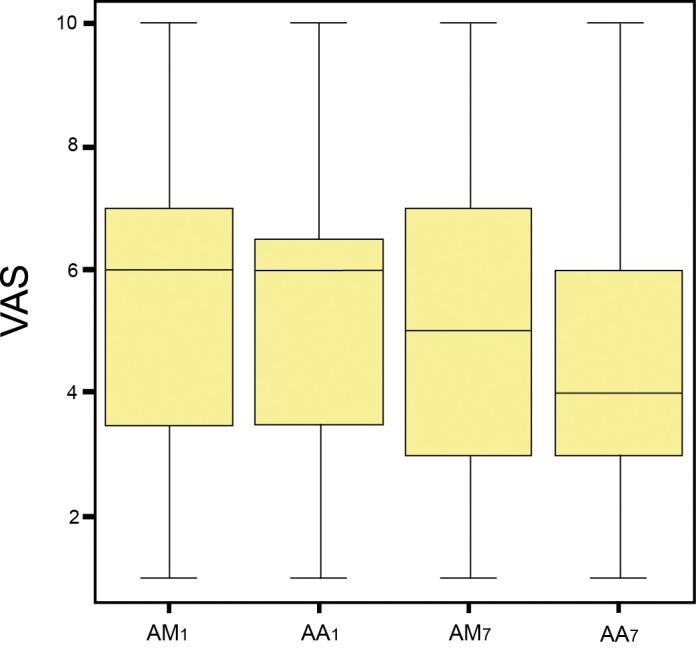
Comparison of the mean visual analog scale (VAS) scores between timepoints during the first week of treatment with the capsaicin rinse (group A): AM1: Baseline score in the morning of the first day of the first week of treatment. AA1: Score in the afternoon of the first day of the week of capsaicin treatment. AM7: Score in the morning of the last day of the week of capsaicin treatment. AA7: Score in the afternoon of the last day of the week of capsaicin treatment.

On examining the VAS scores at the start and end of the first week in both groups (A and B), based on the Wilcoxon test, differences were found between 0.02% capsaicin in the morning (AM1) and afternoon of the first day (AA1) and end of the week (AA7)(Z = -2.31, p=0.003 for AM1 and Z = -2.96, p=0.002 for AA1)(Fig. 1). No such differences were found in the case of the placebo rinse (control group B).

Regarding side effects, intense burning sensation was described by one-third of the subjects during and for a few minutes (maximum 20 minutes) after application of the capsaicin rinse.

## Discussion

Burning sensation affecting the tongue, lips or entire oral cavity may be a symptom of some underlying disorder affecting the oral mucosa, or may be the principal manifestation of a form of idiopathic stomatodynia known as burning mouth syndrome (BMS) (11). The latter is a chronic condition affecting mainly elderly women (as in our own series). In this context, older patients with BMS are known to be more refractory to treatment of the syndrome. A great variety of drugs have been used, though to date no effective management strategy has been established (8).

In our study capsaicin was evaluated in oral rinse formulation, which showed a degree of efficacy in improving the symptoms of BMS over short periods of time. In effect, the visual analog scale (VAS) used to score discomfort revealed significant improvement of at least two points on average after 7 days of treatment with capsaicin rinse versus baseline. No such improvement was observed with the placebo formulation. The patients were recruited on the basis of strict and homogeneous diagnostic criteria. In this sense, many of the studies published to date lack such strict criteria, and the sample characteristics moreover vary from one study to another (3).

Petruzzi et al. (9) also observed evident improvement, but in their case capsaicin was administered via the systemic route during four weeks. As adverse effects, these authors recorded problems such as gastric pain, which limited administration of the drug via this route. Their improvement rate (32%, n = 8) was very similar to our own.

However, it should be noted that there are clear limitations to the use of topical capsaicin, such as limited effect over time and a limited magnitude of improvement (12). While the patients showed improvement from the start to the end of the week of treatment with the active drug formulation, it is also true that the magnitude of such improvement was limited (only 2 points on the VAS on average) – with no complete remission of symptoms in any patient, and no lasting improvement over time.

Moreover, the burning sensation produced on using the capsaicin rinse limits its use – though the discomfort threshold varies among patients (13,14).

Topical capsaicin has been used as a treatment alternative for controlling neuropathic pain in general. The drug is normally used at concentrations of between 0.025% and 0.075%, inducing desensitization to thermal, chemical and mechanical stimuli when applied topically (15).

The mechanism of action involves interaction of capsaicin with the vanilloid receptor (VR1) of the C type sensory nerve fibers (16). These receptors are non-selective cation channels showing high calcium permeability. In this context, capsaicin inhibits the biosynthesis and axonal transport of substance P, a mediator of nociceptive impulses from peripheral stimulation sites towards the central nervous system. Most capsaicin-sensitive fibers terminate in polymodal nociceptors that respond to a broad range of stimuli (heat, pressure and irritants). Topical capsaicin induces selective and reversible desensitization of the afferent sensory C fiber endings. Taking into account that the amyelinic C fibers in the oral mucosa have been implicated in burning sensation in BMS, in the context of the neuropathic hypothesis of the disease (17,18), topical capsaicin should be considered in the management of BMS, though taking into account its limited effect over time and the discomfort caused during use of the oral rinse in one-third of all patients.
